# Educational, but demanding: the experience of therapists in an intensive inpatient trauma treatment program

**DOI:** 10.3389/fpsyg.2025.1581055

**Published:** 2025-08-29

**Authors:** Veronica Vaage-Kowalzik, Jeanette Engeset, Marianne Jakobsen, Julie Horgen Evensen

**Affiliations:** ^1^Division of Mental Health and Addiction, Oslo University Hospital, Oslo, Norway; ^2^Private Practitioner, Asker, Norway

**Keywords:** PTSD, EMDR, PE, therapist rotation, intensive trauma treatment, qualitative research, therapists experience

## Abstract

**Background:**

Intensive trauma treatment programs have shown robust results in treating post-traumatic stress disorder (PTSD). However, the experiences of therapists working within the frameworks of these treatment programs have only scarcely been explored through quantitative studies and have not previously been examined in qualitative studies.

**Objective:**

This study aimed to explore therapists’ experiences in an intensive trauma treatment program (ITTP). Our research questions were: How did the therapist experience working within the ITTP, and how did the treatment program influence them as trauma therapists?

**Methods:**

Seven therapists who had participated in a two-week intensive inpatient trauma treatment program involving Prolonged Exposure, Eye Movement Desensitization and Reprocessing, and therapist rotation (TR) were interviewed using a semi-structured qualitative interview format. The transcripts were analyzed using a thematic analysis approach.

**Results:**

Our analysis resulted in three main themes: (1) Learning through shared experiences, (2) A new experience of trauma therapy changing attitudes and praxis, and (3) Doubts about intensive trauma treatment. The therapists described the treatment project as demanding yet a valuable learning opportunity. Most expressed initial hesitations about providing trauma-focused therapy (TFT), fearing that patients might deteriorate, but participating in the project appeared to alter their attitudes and practices regarding TFT. TR was described as an excellent learning environment and a means to share the burden of responsibility. Our therapists noted that the program’s intensity and multimodality contributed to its effectiveness, though some questioned whether the program was too intensive.

**Conclusion:**

Our findings highlight the uneasiness therapists face when performing TFT and emphasize the value of a supportive and educational atmosphere in delivering TFT. One approach to fostering such an environment is to implement TFT within an intensive treatment program with therapist rotation, allowing therapists to learn from and support one another while providing manualized TFT.

**Clinical trial registration:**

ClinicalTrial.gov identifier: NCT05342480. Date of registration: 2022-04-22.

## 1 Background

During the last 10 years, intensive trauma-focused treatment programs (ITTP) have been developed to better target PTSD symptoms and related reduced vocational and social functioning. These treatment programs target PTSD symptoms using empirically supported trauma-focused treatments including Prolonged Exposure (PE) and Eye Movement Desensitization and Reprocessing (EMDR) detailed in and supported all major PTSD treatment guidelines (American Psychological Association, 2017; Departments of Veterans Affairs and Defense, 2023; International Society for Traumatic Stress Studies, 2018; National Institute for Health Care Excellence, 2018). A systematic review of intensive empirically supported treatments for PTSD indicated that these intensive treatment format have a higher rate of treatment completion compared to non-intensive treatment formats. Intensive treatment was operationalized to refer to treatment delivered more than twice weekly. The review suggests that intensive treatment for PTSD may be an effective alternative to standard treatment to prevent drop-out (Sciarrino et al., 2020). The inpatient treatment program developed at the Psycho-trauma Expertise Center (PSYTREC), Netherlands, is an example of such an intensive trauma-focused treatment (Van Woudenberg et al., 2018). This program includes daily EMDR and PE sessions, physical activity (PA), psychoeducation groups (PEG), as well as therapist rotation (TR) (Van Minnen et al., 2018). The research group has published several studies documenting robust treatment results for patients with PTSD (Van Woudenberg et al., 2018), including those with comorbid disorders (Kolthof et al., 2022; Paridaen et al., 2023) and the dissociative subtype PTSD (Zoet et al., 2018). Other countries have implemented versions of the Dutch intensive treatment program and adapted it to their facilities with good results (Auren et al., 2022; Gahnfelt et al., 2023), and qualitative studies report that patients find the intensity of the treatment and TR important for treatment efficacy (Thoresen et al., 2022; Vaage-Kowalzik et al., 2024).

In sum, there is emerging for evidence for the treatment effect of ITTP. However, little is known about how therapists experience providing trauma-focused therapy (TFT) in an intensive trauma treatment program that includes therapist rotation. Most research to date focus on what hinders the implementation of TFT in a non-intensive treatment models. This body of research shows that many therapists are uncomfortable directly addressing very fear-evoking traumatic memories in treatment sessions (Becker et al., 2004; Grimmett and Galvin, 2015). Therapists have reported fearing that TFT might harm patients and lead to deterioration (Becker et al., 2004; Deacon et al., 2013; Grimmett and Galvin, 2015). Patient-related factors, such as comorbidity (Becker et al., 2004; van Minnen et al., 2010) as well as therapist-related factors, such as negative beliefs or expectations of the consequences of TFT (Farrell et al., 2013; Meyer et al., 2014; van Minnen et al., 2010) are strongly related to the underuse of TFT. In a systematic review of clinicians-related barriers and facilitators to the use of evidence-informed interventions for PTSD, Finch et al. found inflexibility of manualized approaches, fear of creating client distress, working with comorbidities, and lack of training and support to be the most cited barriers to the implementations of evidence-informed interventions (Finch et al., 2020). A recent systematic review of determinants of exposure therapy (ET) in anxiety and PTSD patients found that clinicians’ perceptions of the utility of PE was positively related to ET use. In contrast, negative beliefs about ET was negatively related. Trends in the material suggested that self-efficacy using ET, leadership articulating goals to implement PE, weekly telephone consultations with PE experts, experience treating PTSD, and practical PE training were positively related to ET use (Racz et al., 2024). Factors that limit therapist’s use of TFT in non-intensive treatment settings are likely to be relevant for the providers of TFT in an intensive treatment program, however nuances and differences need to be explored.

To our knowledge, there are no qualitative studies on how therapists experience providing intensive trauma treatment and participating in a therapist rotation treatment program. However, Van Minnen et al. have described and quantitatively reported on two examples of therapist rotation, one of which was similar to our therapist rotation model. In this model, patients met new therapists for their PE and EMDR sessions throughout the treatment program. The therapists held a daily one-hour meeting to discuss progress and treatment plans for the individual patients in their care. The results of the survey indicated that therapist rotation reduced therapists’ fear of conducting TFT with PTSD patients, increased their evaluation of patients’ perceived readiness for TFT, and decreased therapists’ avoidance behavior during sessions (Van Minnen et al., 2018).

Another focus of earlier studies on therapist’s experiences of providing trauma care has been therapist’s mental health and work satisfaction. Mental health professionals are at the risk of experiencing vicarious trauma and burnout (Leung et al., 2023). Finding novel strategies for treatment delivery, and/or making existing methods more supportive of therapists is thus important. Van Minnen et al. suggests that gaining more clinical experience in the safe context of a therapist rotation may lead to better implementation of TFT (Van Minnen et al., 2018). This could imply less risk of therapist burnout at least in the implementation phase, underlining the importance of exploring therapist’s perspectives in the implementation phase of TFT programs.

In the current study, we aimed to explore therapists’ experiences working in a two-week intensive inpatient trauma treatment program (ITTP) that combined EMDR, PE, PA, PEG, and TR. Our research questions were: How did the therapists experience working within the ITTP, and how did the treatment program influence them as trauma therapists?

## 2 Methods

### 2.1 Design and ethics

This study took place in a public psychiatric combined in- and out-patient clinic in Oslo, Norway. The clinic is part of the specialist healthcare system, which requires patients to be referred by a doctor and treats a wide range of mental illnesses. The current study is part of the Norwegian Intensive Inpatient Trauma Treatment Pilot Project. The main goal of this project is to examine whether intensive trauma treatment is feasible in our non-trauma-specialized public healthcare facility.

Eighteen patients participated in the pilot study, divided into three groups of six. Six of these patients also participated in a nested qualitative study (Vaage-Kowalzik et al., 2024).

The Central Norway Regional Ethics Health Committee (REC South East 0704/2022) has approved the study, including the qualitative interviews. ClinicalTrial.gov Identifier NCT05342480. Written informed consent was obtained from all participants.

### 2.2 The patients

Patient inclusion criteria were as follows: fulfilling the diagnostic criteria for a PTSD diagnosis (according to the DSM-IV criteria), >1 traumatic life event, symptoms lasting >6 months, being within the age range of 18 to 65, speaking a Scandinavian language sufficiently, and having undergone at least one prior psychotherapeutic treatment (>3 months duration). Exclusion criteria included having a psychotic or bipolar disorder, an active substance abuse diagnosis, being in a currently abusive or threatening life situation, or having had a serious suicide attempt in the 3 months preceding treatment. Eligibility for the pilot study was determined diagnostically using the Mini-International Neuropsychiatric Interview (MINI) (Sheehan et al., 1998) prior to inclusion by the patients out-patient therapist. Our patient group exhibited high levels of comorbidity, including personality disorders, and also had complex trauma histories involving neglect, bullying, and sexual and physical abuse (Vaage-Kowalzik et al., 2024).

### 2.3 The treatment program

The treatment program had a two-week timeframe with eight full days of treatment. During the mid-treatment weekend, patients returned home. The program included daily PE and EMDR reprocessing sessions, between sessions PA, and PEG, and is described in detail elsewhere (Vaage-Kowalzik et al., 2024). EMDR and Imaginary Exposure (PE) involved daily therapist rotation, allowing patients to meet 11–12 different therapists over the course of the treatment. Therapists and representatives from the ward staff held a daily one-hour meeting to ensure adherence to the treatment PE and EMDR protocols (Foa et al., 2021; Shapiro, 2018) and to monitor progress and plan the following sessions. On-site supervision was provided for PE and EMDR therapy by qualified supervisors to further ensure treatment adherence. All patients remained under the primary care of their outpatient therapists after discharge from the inpatient treatment program. However, therapists were asked to avoid TFT in the 6 months following the completion of treatment and to provide only supportive psychotherapy sessions if necessary.

### 2.4 The therapists

Therapists were interviewed 4–5 months after the second patient group received treatment. Fifteen therapists had participated in the treatment program at the time of the interviews. Seven of these therapists were interviewed for the current study. Of the remaining therapists, five were not interviewed due to their roles in planning and/or supervising the study, and three were not interviewed because they no longer worked at the clinic at the time of the interviews. Participants were approached by email, and no therapist refused participation in the study. Therefore, all available therapists were interviewed for the current study.

The seven therapists interviewed included four PE therapists and three EMDR therapists. Four were psychologists, and three were psychiatrists. The majority of the interviewed therapists were generalists and not trauma treatment specialists, however all had completed courses in either EMDR or PE or both. Their age ranged from 27 to 64 (average 41, mean 41), and they had worked an average of 10 years as therapists (range 4 to 17, mean 9).

An exploratory feasibility study involves fewer participants. The somewhat small sample size was therefore limited by the framework of the project, rather than being a sample of convenience. The sound quality of the interview dialog, the relative homogeneity and specificity of the sample, along with the volume and richness of the data, enhance the information power that the sample holds and permit a smaller sample size (Braun and Clarke, 2022; Malterud et al., 2016). Interviewing seven therapists was thus considered acceptable by the authors.

### 2.5 Data collection

To explore how the therapists experienced working in the intensive trauma treatment program, a semi-structured interview was designed by the study research group. A semi-structured interview ensures both thematic equality between interviews and allows a flexible exploration of the given topics (Alvesson et al., 2020; Kvale and Brinkmann, 2009; Qu and Dumay, 2011). Before the interview, the therapists were informed that they would be asked about their experiences—both positive and negative—while working in the treatment program. The interviewers made an effort to create an encouraging tone, allowing participants to elaborate on relevant themes. The interview guide included eight main questions (see Table 1). Follow-up questions provided participants the opportunity to elaborate on their answers and explore their individual experience of participating as therapists in the program. The interviews lasted 30–45 min and were conducted at the treatment facility. The interviewers were independent of both the treatment program, the treatment ward, and the research project. Three interviewers (one male and two females, in their mid to late thirties, all experienced senior nurses specialized in psychiatry and two with previous experience conducting qualitative research interviews), shared the responsibility, with two present at each interview. Participants were informed that the interviews would be transcribed and anonymized. The sessions were audio recorded and later transcribed and anonymized by a research assistant.

**Table 1:** Examples of questions ask from the interview with therapists.

Topics	Questions
The therapist role	Will you start by briefly describe how you experienced being a therapist in the intensive trauma treatment program? How did you experience the therapist rotation? Has your experience from the intensive trauma treatment program influenced how you are working with trauma patients outside the program?
The treatment program	What do you think may be effective in intensive inpatients trauma treatment? How do you think the treatment components may reduce symptoms and improve function in the patient group? Which trauma patients do you think this treatment program is suited/not suited to? What do you think could be shortcomings in the treatment model? Would you recommend other trauma therapists to participate as therapists in this study?

### 2.6 Analysis

To explore and analyze the material, we employed reflexive thematic analysis (TA) as described by Braun and Clark (Braun and Clarke, 2022). Reflexive TA identifies recurring themes and patterns of meaning-making, allowing for an in-depth exploration and interpretation of data collected from individual interviews. Themes or patterns were identified within the data through an inductive, bottom-up approach, avoiding fitting the data into a pre-existing frame (Patton, 1990). Reflexive TA views “pure” induction as impossible; instead, it acknowledges that researchers bring their philosophical meta-theoretical assumptions and themselves to the analysis, understanding inductive orientation as “grounded” in data (Braun and Clarke, 2022). The authors have different and varied therapeutic orientations. V.V.-K. is a psychodynamic, PE and EMDR therapist, J.H.E. is a cognitive behavioral, emotion-focused and an EMDR therapist, M.J. is an EMDR therapist and certified EMDR supervisor, while J.E. has no specific trauma-therapeutic orientation, but is a certified group-therapist. Two members of the research group hold PhD degrees. This transparency is made in accordance with the checklist of reporting qualitative research by Tong, Sainsbury and Craig (Tong et al., 2007). The author diversity has hopefully broadened and enriched our interpretation of the data. Having a non-trauma therapists in the research group has provided valuable outside perspectives to the discussions, and hopefully aided all authors in approaching the material critically, reducing blind spots that could come when mainly working from one therapeutic orientation. Before commencing the interviews, we speculated about how our therapists would take to therapist rotation. We worried that TR might be too unfamiliar a way of working, possibly giving a sense of lack of control, including little focus on alliance building (bond), which is appreciated as a core element of psychotherapy. As we, as a research and implementation group, had familiarized ourselves with the treatment model through dialog with other ITTP milieus, we were reasonably confident that patients would tolerate the treatment. We perhaps did not fully anticipate how apprehensive some of our therapist sample would be about providing intensive TFT. These preconceptions may have shaped how we structured the interview and approached the data for analysis. Additionally, we have during our analysis of the material been conscious of the fact that the novelty of the treatment and the efforts put into the treatment program could make the therapists more enthusiastic or positive then warranted. We aimed to adjust for this by creating an open and neutral tone in the interview setting, with interviewers independent from the treatment program, that made room for expressing doubt and hesitations.

The analyses were conducted in five phases to increase data analysis transparency (Castleberry and Nolen, 2018). Phase 1: Authors V.V.-K., J.E. and J.H.E. read all transcripts looking for answers to the research questions, explicitly looking for both negative and positive experiences. First and second author generated initial codes and searched for themes independently and later compared and discussed their findings with the last author. To ensure correspondence between raw data and codes specific words and syntax from the transcriptions were used when identifying and labeling various aspects of the data material. Phase 2: Codes were reorganized into possible themes. The last author gave feedback on the first and second author’s reduction and initial thematic categorization further refining the themes and subthemes through discussion. These three authors discussed their unique understanding of the material, and critiqued the categorization as conducted so far. Phase 3: The first, second and last author refined and reorganized both themes and subthemes, comparing them to the initial coded data to ensure our analyzes were consistent with data from the interviews. Phase 4: All authors reviewed listed themes and subthemes and discussed their relevance for the research questions and the research project. The current categorization and presentation were thus agreed upon. This process made our interpretations less dependent on individual preferences (Malterud, 2011). Phase 5: The themes were described in a report that emphasized the essence of the themes. Participants’ quotes were added to support the results and discussion.

We used the labels general, typical, and variant to indicate the recurrence and representativeness of therapists’ experiences as suggested by Hill et al. (2005). When something was mentioned by all, it was labeled as general, and in the text, referred to as “all therapists.” Something was considered typical when it was mentioned by more than half the therapists, in the text referred to as “most therapists.” We use the expression “some therapists” when something was found to be a variant represented by less than half the participants but more than one.

## 3 Results

We identified three main themes in the material; (1) Learning through shared experiences, (2) A new experience of trauma therapy changing attitude and praxis, and (3) Doubts about intensive trauma treatment (see Table 2). All names are pseudonymized.

**Table 2:** The identified themes and subthemes with quotes from the therapists.

Learning through shared experiences	Learning from each other and from the treatment format.	“It was really exciting to get to talk to other therapists, who have more or less experience with different treatment methods. I think I learned a lot.” Hanna
	Togetherness and shared responsibility reduce the burden of patient care.	“It felt good to be a part of a team. To be more therapists handling the same patients.” Susan
	Therapist rotation—benefitting both patient and therapist.	“I think it is a kind of safety measure for the patients. Like a quality enhancement, because they (the patients) get the sum of all of us.” Iris
	Educational, but demanding.	“I think everyone I talked to shared the experience that this was really fun, but also very, very demanding” Hanna
A new experience of trauma therapy changing attitude and praxis	Reframing assumptions about patient capacity.	“What I perhaps have experienced is that patients are able for more than I what I previously maybe feared they were able for.” Camilla
	Braver, less avoidant therapists.	“So now I’m thinking *let us go*, a bit more often than what I maybe used to.” Susan
	Intensity and multiple modalities makes treatment efficient	“I think that it may be effective because it’s so intensive.” Angela
Doubts about intensive trauma treatment	Too intensive?	“… my experience was that it got a bit too intensive. But I think the components in the treatment program are good.” Simone
	Is two weeks enough?	“… many of those that were most severely ill have needed outpatient care afterwards. So, it wasn’t like 2 weeks was enough …” Camilla

### 3.1 Learning through shared experiences

The first theme was termed *learning through shared experiences* and concerned the interviewed therapists’ experiences of the treatment program as a valuable learning opportunity. The therapists expressed how several facets of the program, such as sharing patient care and caseload responsibility, created a sense of safety in their practice. This seemed paramount as the project included patients with complex trauma histories and comorbidity, and it seemed that this experienced safety contributed to the project being an effective learning environment.

#### 3.1.1 Learning from each other within the framework of the treatment format

Most of the therapists described the intensive trauma treatment format, with therapist rotation and daily staff meetings, as a unique possibility to learn from colleagues. Lara said: “… that almost never happens, that so many get involved with the same patient. It is, like, completely extraordinary. So it also makes me think that the treatment gets much better and that you get good input from others. Others have different ways of solving it, so you see that ‘oh, that worked well on that patient, and what I did maybe did not work so well.’ Or the other way around. So you learn a lot from that.” Iris emphasized the benefit of having a very experienced trauma therapist as a part of the therapist group: “It is so seldom that you get the opportunity to work with others on a project, with patients. So that was fun. And that several saw the same patients, and could share experiences. It is really fun to have someone so experienced and an expert like her (names supervisor), who I found very educational to get to work with.”

All therapists reported feeling more proficient and/or confident practicing trauma-focused treatment after having participated in the project. Angela said: “Especially, because at least the first time (working in the program), I wasn’t so steady on PE. So I also thought it was a bit nice to know that there are other people here who are also going to help (treat the patients). Like, then I became a bit calmer, and maybe better at PE, simply because of that. The sort of performance anxiety, to do a good job in the sessions, kind of lessened.”

#### 3.1.2 Togetherness and shared responsibility reduce the burden of patient care

All therapists described close patient care collaboration as positive and that it contributed to a sense of safety in practice. Some emphasized how the shared caseload lessened the burden of individual patient care, and some emphasized the benefit of not being the sole therapists in complicated cases. Susan said: “You did not feel so on your own about the patient (care), like, and you got other perspectives and views on it. It was kind of a bit liberating. That someone else took over this case the next day. You felt you had a bit less responsibility for the patient’s improvement, like. You were kind of part of something bigger.” Hanna put it like this: “I actually thought it was positive for me not to have to work with the same patient for the 2 weeks. And, like because then you were not completely alone with the responsibility if something became difficult or challenging in the treatment process. Like, there were others who had met the patient and could provide thoughts and input.”

#### 3.1.3 Therapist rotation—benefitting both patient and therapist

All the therapists reported positive experiences with TR and said they would like to continue working in this format in the future. TR was described as benefitting both patients and therapists. The therapists said they learned a lot from working closely with colleagues treating the same patients and felt less burdened by their responsibilities when sharing caseloads. (as described in 3.1.1 and 3.1.2). The therapists further described how it appeared that patients benefitting from TR through therapists sharing experiences of what worked for individual patients and working closely together, and from receiving different input from different therapists. Angela described it like this: “I had one (patient) who said she took the best from each therapist, kind of. Like, there was one (therapist) who was very clear and direct, and she took that with her. While another was more caring. So, she managed to pick up different input. For me, if it was difficult, if I had a tough session that I found challenging, it was actually very nice that someone else could see the same patient and see if the same thing happened. So we could kind of discuss X again. So I could get input like ‘yes, but that worked like this,’ or ‘when she dissociates, I found it useful to…’ We could help each other and not just discuss it without actually having felt how it actually was. Like, everyone had had the same patients. So it became much easier to help each other as therapists. And you also feel a bit like you are not as alone with it.”

Some of the therapists also reflected on how TR had changed their view of the role of alliance (bond) between patient and therapist in intensive trauma-focused treatment. Hanna said: “I got the impression that it worked very well, and gave… that the relationship with the therapist was not so important, that it was more the relationship or security in the method, and gaining ownership of their own story that became the important thing. So I think that, I think that was great.” Camilla described how TR—allowed her to both feel more at ease providing TFT and possibly also made it easier to adhere to the treatment manual: “That was the most interesting thing about it (TR) to me, because it was completely new, something I had never done before. Not “owning” a patient alone, and having the responsibility alone, but being able to rest in the process knowing that others were involved, and the fact that you did not get that strong relational connection in the same way as before, probably also made it a bit easier to conduct such strictly manualized treatment.”

Some therapists also described that it can be tough to work in detail with numerous histories of trauma in such a condensed period, but that collaborating closely with other therapists alleviated this burden. Camilla said: “That was the most demanding part of it, to take in so much pain in such a short time. (…) While, as I said, it made it easier to bear because there were many of us. And we had these meetings where we discussed the cases, which for me was crucial to be able to handle it.”

A disadvantage of TR noted by one therapist was the possible disruption of a newly started process. Susan explained: “I mean, you cannot, you cannot continue working on it, if you feel you achieve something in a session, then you might think ‘oh, I should have continued with this.’ You cannot do that. But that’s fine too.”

#### 3.1.4 Educational, but demanding

All the therapists described their involvement in the treatment project as educational. They all stated they would recommend other trauma therapists to participate; however, some expressed reservations, describing how participating as therapists in the treatment program was time consuming and that they, thus, would only recommend participation if tasks outside of the project were reduced or adjusted. Lara said: “So I think that… I think they got very good treatment, and it was fun to see. What wasn’t so great was everything around it, that at the same time—you know, you have patients in other wards, it’s like an already pressing schedule that gets even more pressing.” Camilla said “As such, it was very frustrating as I felt like I did not get enough time to my patients outside the (intensive treatment) program. But if you have the time, I would absolutely recommend it (participating in the program). It was exciting, educational, and incredibly rewarding to be together with so many colleagues working on a few patients.”

### 3.2 A new experience of trauma therapy changing attitude and praxis

The second theme was termed *A new experience of trauma therapy changing attitude and praxis.* This theme cantered on how the intensive treatment program offered therapists novel experiences of trauma-focused treatment. The therapists described experiencing that the patients were able to handle and benefit from the treatment, and that the intensity and multimodality of the program seemed vital for treatment effectiveness. These experiences appeared to influence them as therapists, possibly making them less avoidant and more inclined to offer trauma-focused therapy to a broader range of patients in the future.

#### 3.2.1 Reframing assumptions about patient capacity

All the therapists reported that their views on who could benefit from trauma treatment had changed after participating in the project. Most described experiencing that very symptom-burdened patients can tolerate and benefit from trauma-focused treatment, and further said they thus were more likely to offer trauma therapy to a wider range of patients in the future. Lara said: “It is very inspiring and actually a bit thought-provoking that patients who have struggled so much for a long time can get so much better from intensive treatment. Thus, it has definitely renewed my belief in pretty severely ill patients—that it is worth pushing them quite hard, or challenging them much more than we might have thought we could before.”

Most therapists described previously having worried about patients deteriorating if they were to work therapeutically on details of their trauma history. They, however, described that they had experienced that patients tolerated the treatment. Camilla described: “What I have experienced (in the project) is that patients can handle more than I previously thought. That you can go faster into the worst of the worst, without the patient falling apart. But of course, here (the treatment project) there was also a very, very tight structure. I cannot give that to other patients (outside the project) in the same way. But I think that, at the same time, that maybe I have become a bit tougher going straight into the core of the traumas.”

Having experienced that patients were able to receive intensive trauma-focused treatment, some of the therapists described how they were less inclined to believe that a prolonged stabilization phase was necessary before starting trauma-focused work. Simone put it like this: “…I have a bit more concrete techniques, like, to approach it. That it’s not so dangerous to approach traumas. That it’s a bit of a shift in the attitude toward trauma treatment, in that you do not necessarily have to work so much on stabilization before you dare to go into the trauma.”

Some of the therapists also reflected on the necessity of establishing a close alliance (bond) between the therapist and patient before starting TFT. Angela described how she experienced that the treatment program reduced the role of the alliance between patient and therapist but instead had the advantage of making the patient feel seen by the many therapists and ward staff involved in their treatment: “And I also think they felt very, very invested in. I think that also has something, you might lose something relational, but they get, they become incredibly seen.” Angela further elaborated on the role of the bond between the therapist and the patient, within the framework of the intensive treatment program: “… maybe that relationship does not necessarily have to be completely, like, in place before you start trauma treatment then. That was kind of what we proved a bit. That it should be possible to benefit from processing (traumas) anyway.”

#### 3.2.2 Braver, less avoidant therapists

All therapists in our sample reported that their attitude to and practice of trauma treatment had changed after participating in the project and that they felt more at ease in their role as trauma therapists. Most described that learning from and practicing with others as well as experiencing that patients can tolerate the treatment, has made them more likely to commence TFT, and some described that they were less likely to go along with patients’ avoidance patterns. Susan described how participating in the program had affected how she handled patients avoidance patterns of TFT: “That I may become a bit more focused on not letting the session slip into a lot of other things, which often may happen. Because there are a lot (of issues) surfacing between sessions. And that one kind of instead set aside a large part of the session for some EMDR.” Hanna described how her own avoidance patterns had previously hindered trauma-focused treatment: “I have become, I think I have become less… avoidant… avoided less afterward, in a way. That I am not so afraid to talk about traumas anymore, I am not so afraid that they (the patients) talk about traumas. And that by feeling safe knowing that it’s not dangerous, the patient notices it in a way. Because there have been times before when I have met patients with a lot of traumas where you got a bit scared, you got a bit scared to talk about it almost, because you are afraid they will get worse from it. So I think I have become much less scared of that.”

#### 3.2.3 Intensity and multiple treatment elements seem helpful

All the therapists described experiencing that the intensity of the treatment program and/or the multiple treatment elements was what made the treatment effective. Iris said: “I think it’s the intensity and the combination. That you get to work on it from so many different angles (…) in the, like, PE you get to talk through the different situations. And then you do the EMDR afterwards (…). I think the intensity helps create a real transformation for them (the patients).”

Some therapists, like Iris, described how the treatment elements appeared to complement each other and that PE and EMDR were experienced as good in combination. Other therapists noted that some patients appeared to benefit more from one method than the other, but that the combination still was beneficial. Simone said: “Yes, it… it was interesting that we experienced that some patients benefited greatly from imaginary exposure while others experienced greater benefit from EMDR (…) Also that it was useful to be able to switch between them. That you get a greater effect by having two different approaches to the problem.”

The benefits of inpatient care, with staff available to help patients adhere to the intensive treatment program, was emphasized by some. Hanna said: “I think it worked very well in that way, that the patients received inpatient care, so they kind of always followed the program and attended the sessions.” All described the collaboration between the therapists and between the therapists and ward staff as good, and some emphasized this as important to providing safe and efficient treatment.

### 3.3 Doubts about intensive trauma therapy

The third theme was termed *Doubts about intensive trauma therapy.* This theme highlighted the concerns therapists expressed regarding the intensity and duration of the intensive treatment program. These hesitations were particularly related to the complexity of the patients’ symptoms and trauma histories, as well as worries that post-treatment care might be insufficient.

#### 3.3.1 Too intensive?

As reported above (3.2.3), all interviewed therapists described how the intensity of the treatment program contributed to its effectiveness. At the same time, some of the therapists questioned whether the program was too intensive, with too many treatment elements. Hanna stated, “I actually think that the capacity to learn toward the end of such a day (in treatment) is pretty low. Yeah. So maybe that’s what I’ve thought should possibly be changed—the psychoeducational groups.” Susan expressed concern about whether the program’s intensity could cause patients to begin processing trauma memories they could no longer manage to contain after discharge: “I was just working with EMDR, like. It felt alright, you know. At the same time, I also carry a bit of skepticism. In terms of sort of… intervening and processing, and then suddenly ending. (…) I feel like both methods (EMDR and PE) tend to neglect the patient’s defenses to a great extent. Which can maybe become a bit punishing for them after discharge. Things can get difficult. That you just sort of break-through (the defense) like, and then we work on the difficult stuff. While on the ward, it was alright for those two weeks, but I think maybe for some it became difficult when they went home.”

#### 3.3.2 Is two weeks enough?

Some of the therapists voiced concern about the intensity of the patients’ experiences during the treatment. They suggested that the patients might benefit from a slightly longer treatment program. Simone said: “Also I think maybe we would have benefited from having another week or… to be able to calm things down a bit. Because it became very intense, especially for this particular patient group. Where many of them had complex PTSD or dissociative problems. It became a bit too challenging, I think.” Simone added: “We could have had a bit more time to focus on the sense of mastery after completion. Like, it went so quickly, from feeling they had mastered it (the program) to (phew) having to leave the ward again.”

Some therapists emphasized that the participating patients experienced PTSD from early traumas, neglect, and poor attachment patterns, along with other comorbid psychiatric disorders. They noted that the complexity and duration of these traumas likely necessitate longer-term psychiatric treatment with more specified outpatient follow-up after the intensive trauma treatment program. Camilla said: “I also think that would be to underestimate the difficulties they (the patients) actually have, to believe that two weeks would be enough. Many have experienced traumas throughout their lives…. More like attachment traumas. So that’s maybe one of the things I thought about the most afterwards, that there the follow-up afterwards should be better planned for, for many of them.”

## 4 Discussion

This study aimed to explore how therapists in a Norwegian state-funded psychiatric centre experienced working within an intensive trauma-focused treatment program, and how the treatment program influenced them as trauma therapists. The therapists in our study described participating in the project as a great learning experience. They did, however, experience it as demanding and emphasized the need to facilitate and make adjustments to their workload outside the treatment program in the weeks the project was running. The therapists described how the ITTP had altered their attitude to and practice of TFT. Experiencing first-hand that PTSD patients with complex trauma histories and comorbidities were able to tolerate intensive treatment seems to have made them more secure in their role as trauma therapists, and possibly more likely to provide TFT in the future. Therapist rotation was described a great learning arena and a way of sharing the burden of responsibility. Our therapists described the intensity and multimodality of the program as effective, but some questioned if the program was too intensive, and if some patients would be better served by a slightly longer, less intensive program, or a tighter follow-up program. The results will now be discussed in detail.

### 4.1 What makes therapists equipped and ready to provide TFT?

Similar to findings reported in earlier literature (Finch et al., 2020), our therapists expressed concern about providing TFT to patients with complex trauma histories and comorbidities, fearing that patients might not tolerate the treatment and could deteriorate. The stress-inducing potential of delivering exposure treatment is effectively illustrated by a study showing how novice therapists experienced high levels of stress, both physiologically (cortisol) and psychologically, at the start of *in vivo* exposure sessions with anxiety-disordered patients (Schumacher et al., 2017). The study also found that stress levels decreased both within and across three exposure sessions. This parallels our therapists’ expressed relief when they report to experience that patients can tolerate and benefit from TFT. Furthermore, most of our therapists have been educated in the three-phase model of trauma treatment (Cloitre et al., 2011; Herman, 1992), where the first phase—stabilization—is viewed as essential before initiating TFT. This misconception about TFT has been noted as a reason some therapists delay starting effective treatment (Murray et al., 2022). So, what encouraged our therapists to provide TFT to patients with PTSD and comorbidities?

All our therapists received training in a specific form of TFT, which has been found to correlate with the use of ET (Racz et al., 2024). Furthermore, we found that sharing the responsibility for patient treatment and progress seemed to alleviate the burden on individual therapists, making it easier to initiate TFT. In the three-phase trauma treatment model, establishing an alliance (bond) is central to the stabilization phase and is regarded as a prerequisite for TFT (Cloitre et al., 2011). In the current study, however, no such bond with individual therapists was formed either before or during treatment. Some of our therapists expressed how their belief that a long-established alliance (bond) between patient and therapist was necessary for conducting TFT had changed. In an earlier study examining patients’ experiences of ITTP, we reported that patients appeared to develop an alliance (bond) with the multidisciplinary team and ward staff, as well as a working alliance with the rotating therapists and the treatment program itself, rather than forming an individual bond with a therapist (Vaage-Kowalzik et al., 2024). This aligns with findings from a previously described quantitative study, which indicated that even patients with attachment problems could foster a strong working alliance in an intensive trauma treatment program with TR (Van Minnen et al., 2018). Our results may suggest that therapists also perceive the absence of an emotional bond as relieving, as it further alleviates the weight of experienced responsibility. This could be viewed as detrimental to the treatment process if it implies that therapists become less orientated toward providing high quality patient care. Our sample of therapists does appear to be invested in the individual patients in the treatment program. However, how sharing patient load effects therapists continually working in intensive treatment programs warrant investigation. Furthermore, a diminished emphasis on the emotional bond might potentially afford patients greater ownership of their trauma treatment process. This was explicitly articulated by a patient in our earlier study, who noted that collaborating with several therapists made her feel that the therapy process became her own, rather than a shared experience with a therapist (Vaage-Kowalzik et al., 2024).

In our earlier study of patients’ experiences with ITTP, we found that the patients were also worried about entering the ITTP and that it was crucial for them to feel safe within the framework of the treatment program. The patients described how access to ward staff, a supportive multidisciplinary team, and the pretreatment admission made them feel safe, emphasizing this as beneficial to the treatment process. While most of our therapists did not report that these features provided them with a sense of safety when conducting TFT, some highlighted the importance of ward staff, inpatient care, and effective collaboration between staff and therapists in delivering safe and efficient treatment.

### 4.2 Therapist rotation – particularly useful in TFT?

All the therapists in our sample were positive to TR. The TR model used in our project was designed to share the burden of responsibility of patient care (Van Minnen et al., 2018). This effect was described by all therapists in our study. Most therapies involve exposing oneself to emotional pain and challenge the capacity to regulate overwhelming emotional states. TFT is, however, extraordinary as it aims to work in-depth with the most painful and vulnerable episodes of a patient’s history often exposing gruesome details of abuse and neglect. As documented in previous research and further described in the current study, therapists’ anxiety related to TFT is common. The fear of patient deterioration, of causing harm, and of having the sole responsibility for patient care may stop therapists from providing TFT and hinder patients from availing of treatment that may benefit them. TR may thus be particularly useful in TFT.

The TR model was, furthermore, designed to reduce hindrances to TFT implementation including therapist avoidance and drift, and a quantitative survey found that therapists reported that they were less likely to drift from the treatment protocol and were less fearful of pressing ahead in TFT sessions (Van Minnen et al., 2018). Providing manualized treatment was not a topic emphasized by our sample of therapists, however, one therapist described how the decreased focus on alliance in the TR model, in combination with the shared responsibility of patient care made it easier to adhere to the therapy manual. The therapist group furthermore reported that participating in the program had changed their attitude to and practice of TFT, and some described being less likely to go along with patients’ avoidance patterns, limiting avoidance and drift.

Creating effective therapist training is challenging, and the gold standard is often taken to be supervision where therapists bring video recordings of their therapy sessions (Haggerty and Hilsenroth, 2011). Our interviewed therapists differed greatly in clinical experience, from novice to 17 years of experience. Our therapist group furthermore included a senior EMDR therapist (not interviewed in this study) with more than 30 years of experience working and supervising TFT. The TR model with daily meetings where individual patients’ progress was discussed provided an extraordinary form of supervision as the supervisor not only knew the patients in-depth but also gained experience treating them. Many trauma treatment facilities do not have a trauma treatment supervisor on site who is available to contribute on a therapist rotation team. This form of master-apprentice learning may, however, be provided by more experienced members of the treatment team. The benefit of supervision is mentioned explicitly by one of our therapists and the program structure with daily meetings was emphasized by others. All our therapists furthermore reported that participating in the treatment program had benefitted their praxis as trauma therapists, most describing that they would be more likely to commence TFT in patients with complex histories of trauma in the future.

TR may also be particularly useful in TFT as the daily meetings provide an arena to support each other and share experiences of providing TFT to a shared caseload of patients. Therapist burnout is well documented in the trauma therapist population (Craig and Sprang, 2010; Sodeke-Gregson et al., 2013), and has been shown to be associated with lower odds of clinical improvement in PTSD symptoms in patients receiving TFT (Sayer et al., 2024). TR might lessen the burden on individual therapists and create a sheltering environment that possibly could reduce the risk of burnout and therapists leaving the workforce. On the other hand, providing trauma care in to severely symptom burdened patients in an intensive format could constitute a stressor that daily staff meetings, supervison and shared caseloads may not mitigate. Not being in a therapeutic framework that allows for the formation of an alliance (bond) may also be experienced as a toll for trauma therapists for whom working to create a bond with their patients have been an integral part of their earlier training and praxis.

### 4.3 What’s the right level of intensity and length of treatment?

All our therapists described how the intensity of the treatment program was part of what made it effective. However, some of the therapists wondered if the program was too intensive, with too many treatment elements. Some therapists also emphasized the complexity and duration of the participating patients’ traumas, as well as their burden of comorbidities, and reasoned that this made the patients likely to require longer-term psychiatric treatment with more specified outpatient follow-up after discharge. As detailed in the introduction, quantitative research has shown robust effects of ITTP. However, this treatment has also shown a stratified response where patients can be grouped into fast, slow, partial, and non-responders (Hendriks et al., 2018). Lately, more focus has been directed toward finding predictors and moderators of treatment outcomes to better understand what part of the PTSD population profit from TFT and what subsamples might need other, adjusted, or adjunctive treatment (van Vliet et al., 2018). In a twice-weekly EMDR-based TFT program for PTSD resulting from childhood abuse, high levels of pretreatment PTSD symptoms were the only identified predictor of a less effective treatment outcome. The authors suggested that patients with severe PTSD symptoms may benefit from additional sessions or the incorporation of other evidence-based treatment approaches. Adding a stabilization program to TFT has been explored as an option that could possibly benefit partial or non-responders. An RCT comparing twice-weekly EMDR to EMDR preceded by an eight-session stabilization program has, however, shown EMDR without stabilization to be non-inferior to EMDR preceded by stabilization (van Vliet et al., 2018), and a similar RCT using PE shows comparable results (Oprel et al., 2021).

The therapists in our study are concerned about how patients will fare after the treatment program and question whether patients need more tailored follow-up care. This supports the findings of our previous study on patients’ experiences with ITTP, in which most of our sample described the transition from two intensive weeks of treatment back home as challenging and expressed a wish to have been better prepared for discharge (Vaage-Kowalzik et al., 2024). Could it be that some patients, particularly partial or slow responders, would benefit from a modified stabilization and consolidation program after treatment? This is consistent with Herman’s original description of the phases of recovery as “oscillating and dialectical in nature” (Herman, 1992), where trauma treatment revolves around and returns to issues of safety and trust, remembrance and mourning, as well as consolidation and reconnection.

### 4.4 Strengths and limitations

This study has some important limitations, including the sample size. However, the somewhat small number of therapists interviewed in our study is offset by the in-depth format of our interviews, which allows for exploring a range of participants’ experiences. Furthermore, the novel treatment intervention examined in this study has been explored qualitatively only to a limited extent, and therapists’ experiences have not been investigated qualitatively before. In a treatment model that includes therapist rotation—a radically new approach to delivering trauma-focused therapy—qualitative research into both patients and therapists is crucial. Another possible limitation is that all interviewed therapists were women, which may restrict transferability. The interviews were conducted some time after the completion of the first two treatment programs, so the therapists retained clear memories of their participation. However, not enough time had necessarily elapsed to assess the long-term impact on the therapists or how the program influenced them in treating future trauma patients. Furthermore, positive statements from the therapists about their impressions of treatment effects need obviously be supported by quantitative, and further qualitative data.

Our intensive trauma treatment program occurred in a facility not specialized in trauma treatment, and our therapists and staff were primarily non-expert trauma therapists. This could limit the effectiveness of the treatment program and may also mean that our sample of therapists was more uneasy about providing TFT than therapists in a more specialized trauma center. However, our therapist sample possesses good ecological validity, and their experiences could be valuable as more non-trauma-specialized centers seek to implement both intensive and non-intensive TFT.

## 5 Conclusions and recommendations

In line with earlier literature on clinician-related barriers to providing trauma-focused treatment, our therapist sample highlights the toll of working with patients’ complex trauma histories, including the expressed fear of causing deterioration. Practicing in an intensive trauma treatment format with therapist rotation appears to have lessened the therapists’ burden through shared responsibility for patient care. Experiencing firsthand that even patients with comorbidities and complex trauma histories can tolerate TFT seems to empower therapists, potentially making them more likely to provide TFT in the future. Therefore, intensive trauma treatment programs appear beneficial for patients and offer a solid and supportive training environment for therapists.

Clinical implications: Our findings indicate that clinicians’ anxieties about providing TFT should be acknowledged, as these concerns may impede the adoption of evidence-based, trauma-focused treatment. Encouraging novice therapists to practice TFT in supportive settings with shared patient care may increase their adherence to treatment protocols and reduce the likelihood of drift.

Future research recommendations: Some of our therapists described how EMDR and PE complement each other and work well in combination. In our earlier study, we reported that some patients described a seemingly synergistic effect between the treatment modalities, with PE and EMDR complementing each other to strengthen the combined effect (Vaage-Kowalzik et al., 2024). A possible synergism from combining EMDR and PE has not been explicitly investigated in research so far and should be explored in quantitative studies. Future research should furthermore explore the possible benefits of using TR as an arena for supervision and skills training.

## Data Availability

The raw data supporting the conclusions of this article will be made available by the authors, without undue reservation.
